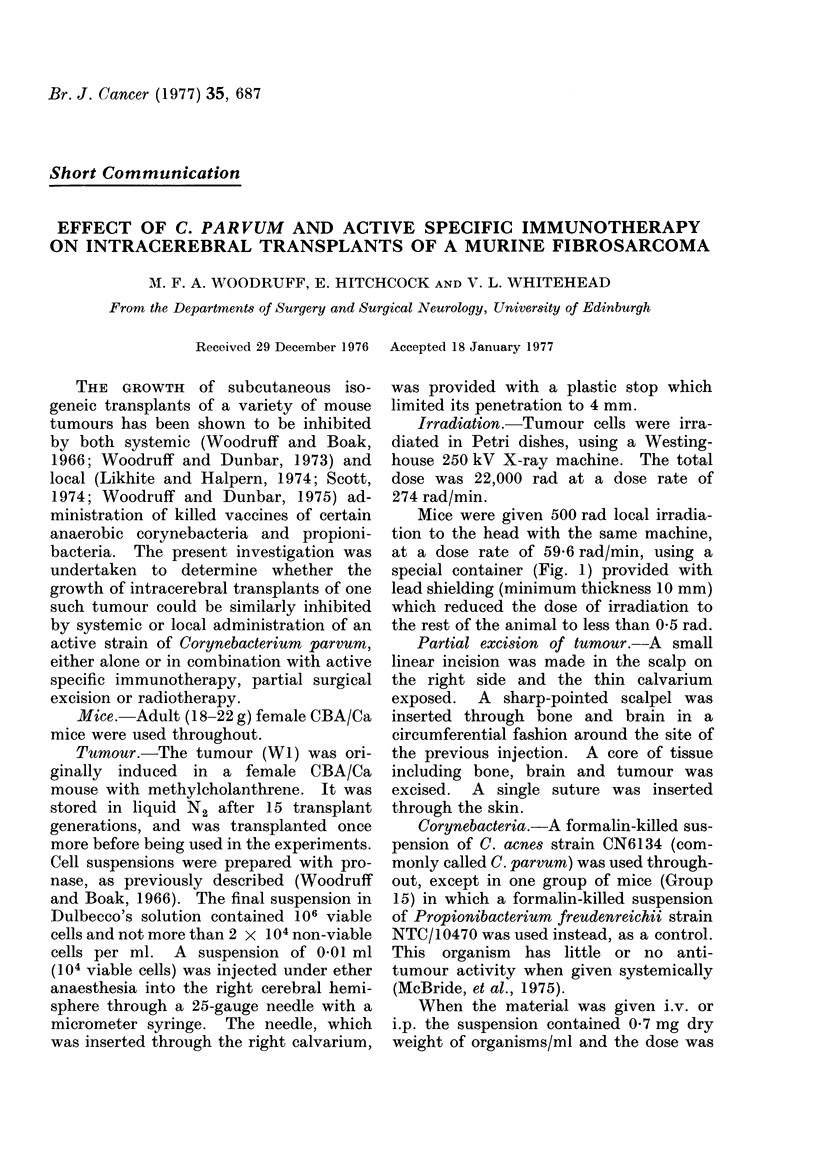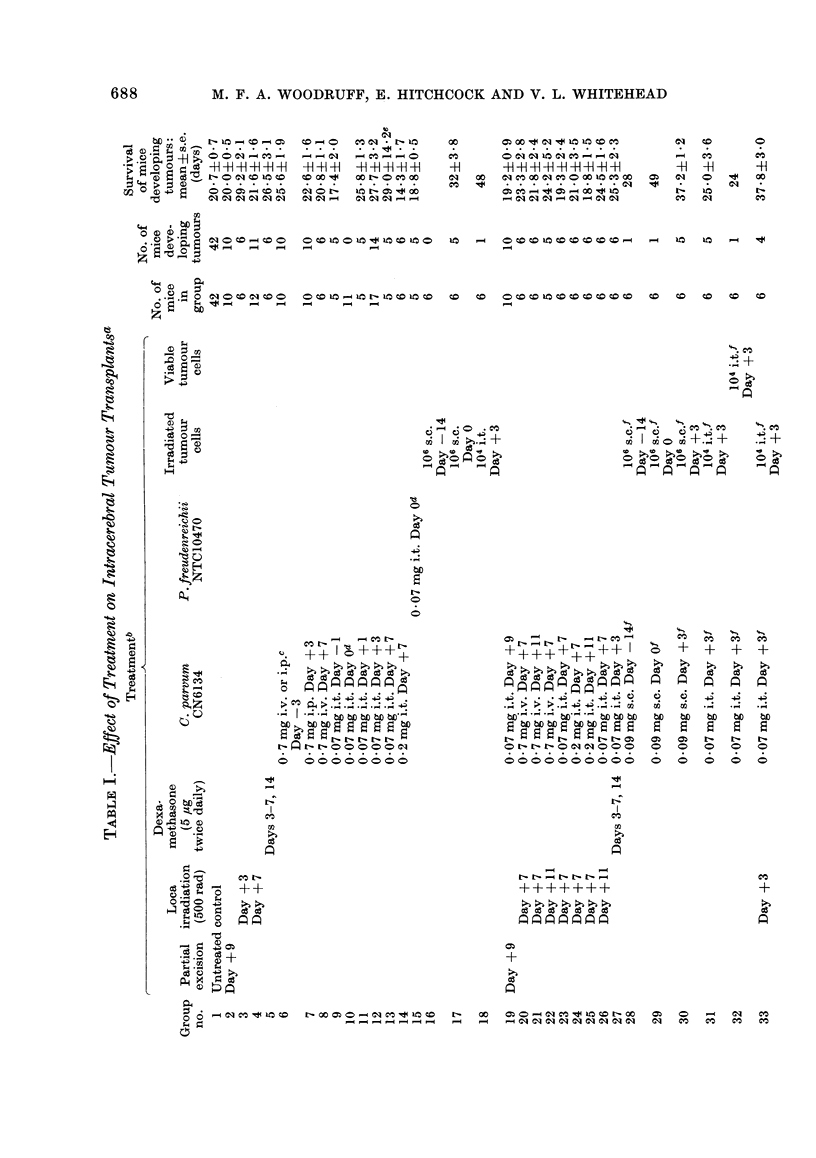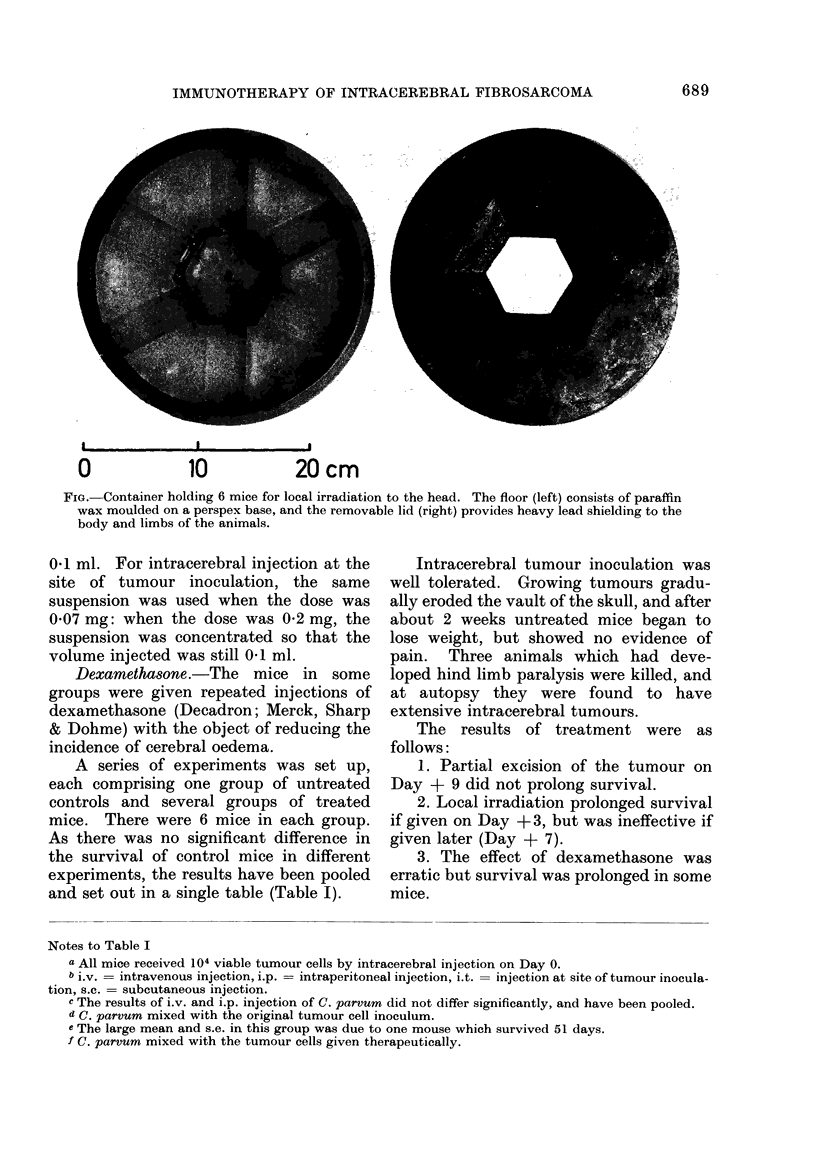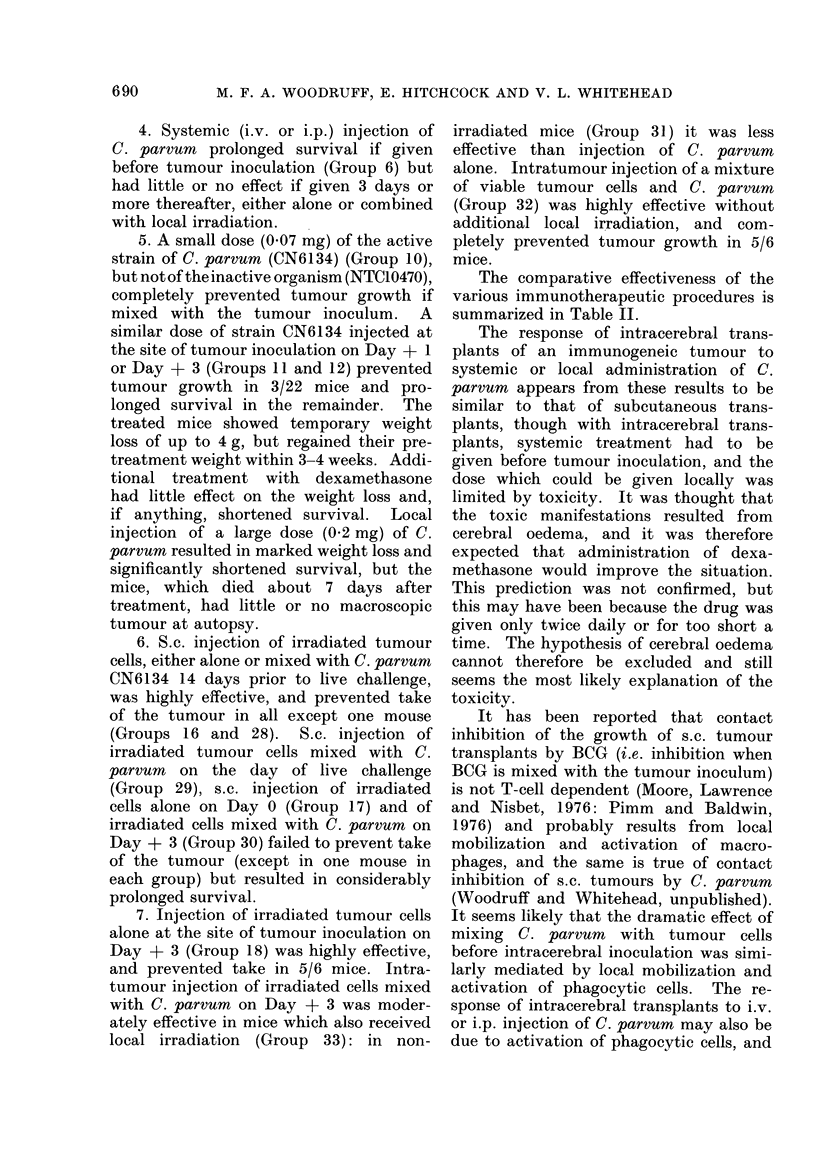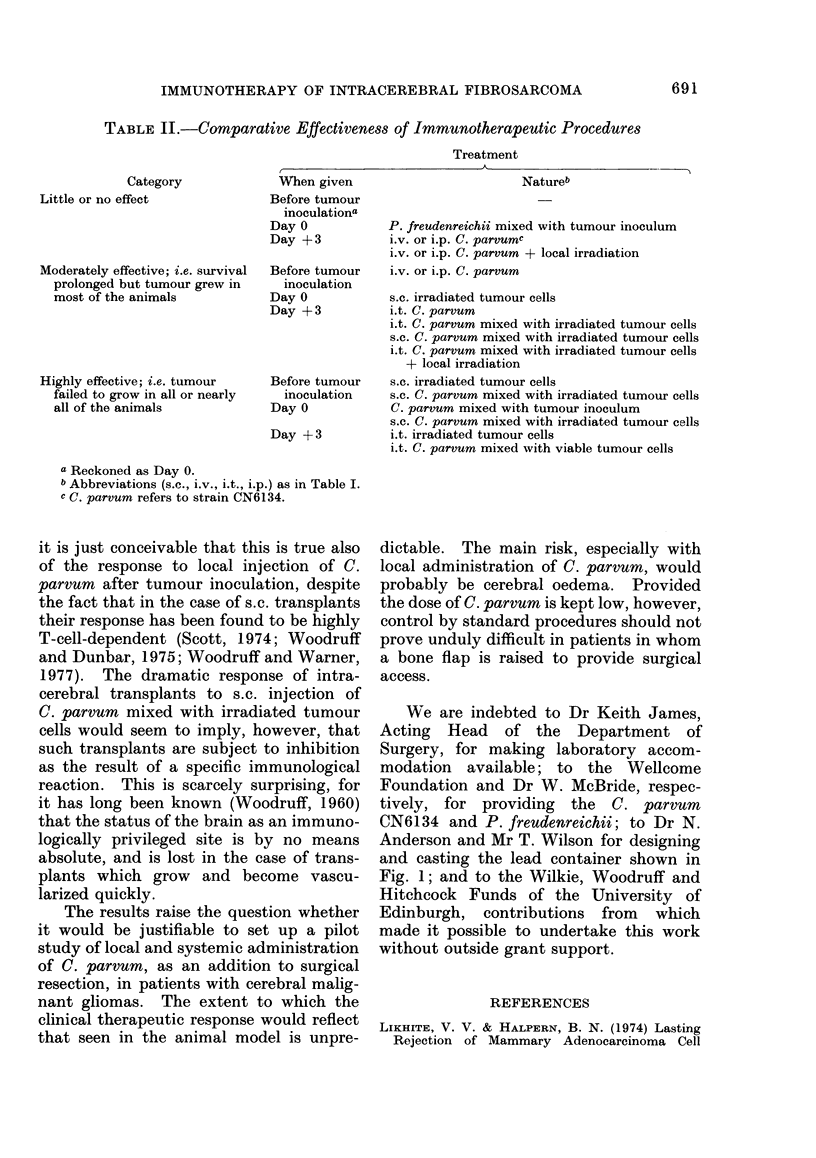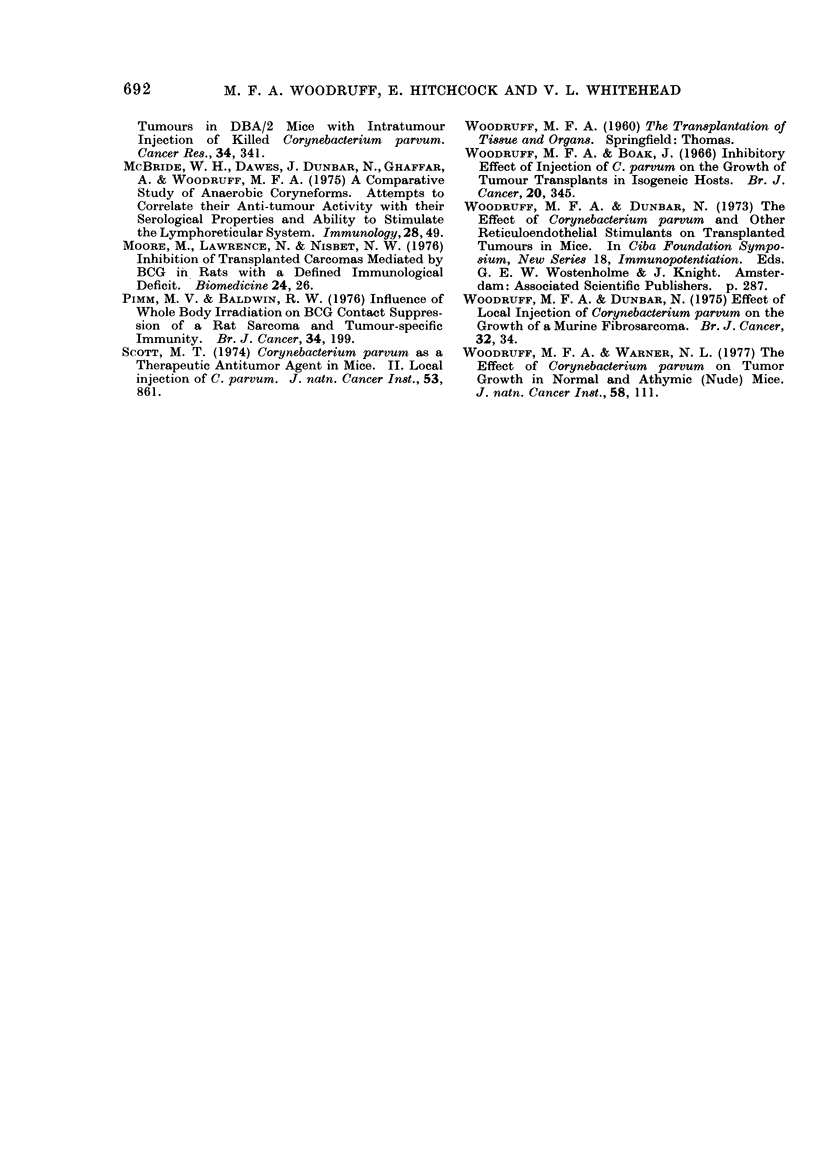# Effect of C. parvum and active specific immunotherapy on intracerebral transplants of a murine fibrosarcoma.

**DOI:** 10.1038/bjc.1977.105

**Published:** 1977-05

**Authors:** M. F. Woodruff, E. Hitchcock, V. L. Whitehead

## Abstract

**Images:**


*Br. J. Cancer* (1977) **35**, 687

***Short Communication***

### EFFECT OF *C. PARVUM* AND ACTIVE SPECIFIC IMMUNOTHERAPY ON INTRACEREBRAL TRANSPLANTS OF A MURINE FIBROSARCOMA

M. F. A. WOODRUFF, E. HITCHCOCK AND V. L. WHITEHEAD

*From the Departments of Surgery and Surgical Neurology, University of Edinburgh*

Received 29 December 1976 Accepted 18 January 1977

THE GROWTH of subcutaneous isogeneic transplants of a variety of mouse tumours has been shown to be inhibited by both systemic (Woodruff and Boak, 1966; Woodruff and Dunbar, 1973) and local (Likhite and Halpern, 1974; Scott, 1974; Woodruff and Dunbar, 1975) administration of killed vaccines of certain anaerobic corynebacteria and propionibacteria. The present investigation was undertaken to determine whether the growth of intracerebral transplants of one such tumour could be similarly inhibited by systemic or local administration of an active strain of *Corynebacterium parvum*, either alone or in combination with active specific immunotherapy, partial surgical excision or radiotherapy.

*Mice*.—Adult (18–22 g) female CBA/Ca mice were used throughout.

*Tumour*.—The tumour (W1) was originally induced in a female CBA/Ca mouse with methylcholanthrene. It was stored in liquid $N_2$ after 15 transplant generations, and was transplanted once more before being used in the experiments. Cell suspensions were prepared with pronase, as previously described (Woodruff and Boak, 1966). The final suspension in Dulbecco's solution contained $10^6$ viable cells and not more than $2 \times 10^4$ non-viable cells per ml. A suspension of 0·01 ml ($10^4$ viable cells) was injected under ether anaesthesia into the right cerebral hemisphere through a 25-gauge needle with a micrometer syringe. The needle, which was inserted through the right calvarium, was provided with a plastic stop which limited its penetration to 4 mm.

*Irradiation*.—Tumour cells were irradiated in Petri dishes, using a Westinghouse 250 kV X-ray machine. The total dose was 22,000 rad at a dose rate of 274 rad/min.

Mice were given 500 rad local irradiation to the head with the same machine, at a dose rate of 59·6 rad/min, using a special container (Fig. 1) provided with lead shielding (minimum thickness 10 mm) which reduced the dose of irradiation to the rest of the animal to less than 0·5 rad.

*Partial excision of tumour*.—A small linear incision was made in the scalp on the right side and the thin calvarium exposed. A sharp-pointed scalpel was inserted through bone and brain in a circumferential fashion around the site of the previous injection. A core of tissue including bone, brain and tumour was excised. A single suture was inserted through the skin.

*Corynebacteria*.—A formalin-killed suspension of *C. acnes* strain CN6134 (commonly called *C. parvum*) was used throughout, except in one group of mice (Group 15) in which a formalin-killed suspension of *Propionibacterium freudenreichii* strain NTC/10470 was used instead, as a control. This organism has little or no antitumour activity when given systemically (McBride, *et al.*, 1975).

When the material was given i.v. or i.p. the suspension contained 0·7 mg dry weight of organisms/ml and the dose was

TABLE I.—*Effect of Treatment on Intracerebral Tumour Transplants*[a]

| Group no. | Treatment[b]: Partial excision | Treatment[b]: Loca irradiation (500 rad) | Treatment[b]: Dexa-methasone (5 μg twice daily) | Treatment[b]: *C. parvum* CN6134 | Treatment[b]: *P. freudenreichii* NTC10470 | Treatment[b]: Irradiated tumour cells | Treatment[b]: Viable tumour cells | No. of mice in group | No. of mice deve-loping tumours | Survival of mice developing tumours: mean ± s.e. (days) |
|---|---|---|---|---|---|---|---|---|---|---|
| 1 | Untreated control | | | | | | | 42 | 42 | 20·7 ± 0·7 |
| 2 | Day +9 | | | | | | | 10 | 10 | 20·0 ± 0·5 |
| 3 | | Day +3 | | | | | | 6 | 6 | 29·2 ± 2·1 |
| 4 | | Day +7 | | | | | | 12 | 11 | 21·6 ± 1·6 |
| 5 | | | Days 3–7, 14 | | | | | 6 | 6 | 26·5 ± 3·1 |
| 6 | | | | 0·7 mg i.v. or i.p.[c] Day −3 | | | | 10 | 10 | 25·6 ± 1·9 |
| 7 | | | | 0·7 mg i.p. Day +3 | | | | 10 | 10 | 22·6 ± 1·6 |
| 8 | | | | 0·7 mg i.v. Day +7 | | | | 6 | 6 | 20·8 ± 1·1 |
| 9 | | | | 0·07 mg i.t. Day −1 | | | | 5 | 5 | 17·4 ± 2·0 |
| 10 | | | | 0·07 mg i.t. Day 0[d] | | | | 11 | 0 | |
| 11 | | | | 0·07 mg i.t. Day +1 | | | | 5 | 5 | 25·8 ± 1·3 |
| 12 | | | | 0·07 mg i.t. Day +3 | | | | 17 | 14 | 27·7 ± 3·2 |
| 13 | | | | 0·07 mg i.t. Day +7 | | | | 5 | 5 | 29·0 ± 14·2[e] |
| 14 | | | | 0·2 mg i.t. Day +7 | | | | 6 | 6 | 14·3 ± 1·7 |
| 15 | | | | | 0·07 mg i.t. Day 0[d] | | | 5 | 5 | 18·8 ± 0·5 |
| 16 | | | | | | $10^6$ s.c. Day −14 | | 6 | 0 | |
| 17 | | | | | | $10^6$ s.c. Day 0 | | 6 | 5 | 32 ± 3·8 |
| 18 | | | | | | $10^4$ i.t. Day +3 | | 6 | 1 | 48 |
| 19 | Day +9 | | | 0·07 mg i.t. Day +9 | | | | 10 | 10 | 19·2 ± 0·9 |
| 20 | | Day +7 | | 0·7 mg i.v. Day +7 | | | | 6 | 6 | 23·3 ± 2·8 |
| 21 | | Day +7 | | 0·7 mg i.v. Day +11 | | | | 6 | 6 | 21·8 ± 2·4 |
| 22 | | Day +11 | | 0·7 mg i.v. Day +7 | | | | 5 | 5 | 24·2 ± 5·2 |
| 23 | | Day +7 | | 0·07 mg i.t. Day +7 | | | | 6 | 6 | 19·3 ± 2·4 |
| 24 | | Day +7 | | 0·2 mg i.t. Day +7 | | | | 6 | 6 | 21·0 ± 3·5 |
| 25 | | Day +7 | | 0·2 mg i.t. Day +11 | | | | 6 | 6 | 18·8 ± 1·5 |
| 26 | | Day +11 | | 0·07 mg i.t. Day +7 | | | | 6 | 6 | 24·5 ± 1·6 |
| 27 | | | Days 3–7, 14 | 0·07 mg i.t. Day +3 | | | | 6 | 6 | 25·3 ± 2·3 |
| 28 | | | | 0·09 mg s.c. Day −14[f] | | $10^6$ s.c.[f] Day −14 | | 6 | 1 | 28 |
| 29 | | | | 0·09 mg s.c. Day 0[f] | | $10^6$ s.c.[f] Day 0 | | 6 | 1 | 49 |
| 30 | | | | 0·09 mg s.c. Day +3[f] | | $10^6$ s.c.[f] Day +3 | | 6 | 5 | 37·2 ± 1·2 |
| 31 | | | | 0·07 mg i.t. Day +3[f] | | $10^4$ i.t.[f] Day +3 | | 6 | 5 | 25·0 ± 3·6 |
| 32 | | | | 0·07 mg i.t. Day +3[f] | | | $10^4$ i.t.[f] Day +3 | 6 | 1 | 24 |
| 33 | | Day +3 | | 0·07 mg i.t. Day +3[f] | | $10^4$ i.t.[f] Day +3 | | 6 | 4 | 37·8 ± 3·0 |

FIG.—Container holding 6 mice for local irradiation to the head. The floor (left) consists of paraffin wax moulded on a perspex base, and the removable lid (right) provides heavy lead shielding to the body and limbs of the animals.

0·1 ml. For intracerebral injection at the site of tumour inoculation, the same suspension was used when the dose was 0·07 mg: when the dose was 0·2 mg, the suspension was concentrated so that the volume injected was still 0·1 ml.

*Dexamethasone*.—The mice in some groups were given repeated injections of dexamethasone (Decadron; Merck, Sharp & Dohme) with the object of reducing the incidence of cerebral oedema.

A series of experiments was set up, each comprising one group of untreated controls and several groups of treated mice. There were 6 mice in each group. As there was no significant difference in the survival of control mice in different experiments, the results have been pooled and set out in a single table (Table I).

Intracerebral tumour inoculation was well tolerated. Growing tumours gradually eroded the vault of the skull, and after about 2 weeks untreated mice began to lose weight, but showed no evidence of pain. Three animals which had developed hind limb paralysis were killed, and at autopsy they were found to have extensive intracerebral tumours.

The results of treatment were as follows:

1. Partial excision of the tumour on Day + 9 did not prolong survival.
2. Local irradiation prolonged survival if given on Day +3, but was ineffective if given later (Day + 7).
3. The effect of dexamethasone was erratic but survival was prolonged in some mice.

---

Notes to Table I

[a] All mice received $10^4$ viable tumour cells by intracerebral injection on Day 0.

[b] i.v. = intravenous injection, i.p. = intraperitoneal injection, i.t. = injection at site of tumour inoculation, s.c. = subcutaneous injection.

[c] The results of i.v. and i.p. injection of *C. parvum* did not differ significantly, and have been pooled.

[d] *C. parvum* mixed with the original tumour cell inoculum.

[e] The large mean and s.e. in this group was due to one mouse which survived 51 days.

[f] *C. parvum* mixed with the tumour cells given therapeutically.

4. Systemic (i.v. or i.p.) injection of *C. parvum* prolonged survival if given before tumour inoculation (Group 6) but had little or no effect if given 3 days or more thereafter, either alone or combined with local irradiation.

5. A small dose (0·07 mg) of the active strain of *C. parvum* (CN6134) (Group 10), but not of the inactive organism (NTC10470), completely prevented tumour growth if mixed with the tumour inoculum. A similar dose of strain CN6134 injected at the site of tumour inoculation on Day + 1 or Day + 3 (Groups 11 and 12) prevented tumour growth in 3/22 mice and prolonged survival in the remainder. The treated mice showed temporary weight loss of up to 4 g, but regained their pre-treatment weight within 3–4 weeks. Additional treatment with dexamethasone had little effect on the weight loss and, if anything, shortened survival. Local injection of a large dose (0·2 mg) of *C. parvum* resulted in marked weight loss and significantly shortened survival, but the mice, which died about 7 days after treatment, had little or no macroscopic tumour at autopsy.

6. S.c. injection of irradiated tumour cells, either alone or mixed with *C. parvum* CN6134 14 days prior to live challenge, was highly effective, and prevented take of the tumour in all except one mouse (Groups 16 and 28). S.c. injection of irradiated tumour cells mixed with *C. parvum* on the day of live challenge (Group 29), s.c. injection of irradiated cells alone on Day 0 (Group 17) and of irradiated cells mixed with *C. parvum* on Day + 3 (Group 30) failed to prevent take of the tumour (except in one mouse in each group) but resulted in considerably prolonged survival.

7. Injection of irradiated tumour cells alone at the site of tumour inoculation on Day + 3 (Group 18) was highly effective, and prevented take in 5/6 mice. Intratumour injection of irradiated cells mixed with *C. parvum* on Day + 3 was moderately effective in mice which also received local irradiation (Group 33): in non-irradiated mice (Group 31) it was less effective than injection of *C. parvum* alone. Intratumour injection of a mixture of viable tumour cells and *C. parvum* (Group 32) was highly effective without additional local irradiation, and completely prevented tumour growth in 5/6 mice.

The comparative effectiveness of the various immunotherapeutic procedures is summarized in Table II.

The response of intracerebral transplants of an immunogeneic tumour to systemic or local administration of *C. parvum* appears from these results to be similar to that of subcutaneous transplants, though with intracerebral transplants, systemic treatment had to be given before tumour inoculation, and the dose which could be given locally was limited by toxicity. It was thought that the toxic manifestations resulted from cerebral oedema, and it was therefore expected that administration of dexamethasone would improve the situation. This prediction was not confirmed, but this may have been because the drug was given only twice daily or for too short a time. The hypothesis of cerebral oedema cannot therefore be excluded and still seems the most likely explanation of the toxicity.

It has been reported that contact inhibition of the growth of s.c. tumour transplants by BCG (*i.e.* inhibition when BCG is mixed with the tumour inoculum) is not T-cell dependent (Moore, Lawrence and Nisbet, 1976: Pimm and Baldwin, 1976) and probably results from local mobilization and activation of macrophages, and the same is true of contact inhibition of s.c. tumours by *C. parvum* (Woodruff and Whitehead, unpublished). It seems likely that the dramatic effect of mixing *C. parvum* with tumour cells before intracerebral inoculation was similarly mediated by local mobilization and activation of phagocytic cells. The response of intracerebral transplants to i.v. or i.p. injection of *C. parvum* may also be due to activation of phagocytic cells, and

TABLE II.—*Comparative Effectiveness of Immunotherapeutic Procedures*

| Category | Treatment: When given | Treatment: Nature[b] |
|---|---|---|
| Little or no effect | Before tumour inoculation[a] | — |
| | Day 0 | *P. freudenreichii* mixed with tumour inoculum |
| | Day +3 | i.v. or i.p. *C. parvum*[c] |
| | | i.v. or i.p. *C. parvum* + local irradiation |
| Moderately effective; *i.e.* survival prolonged but tumour grew in most of the animals | Before tumour inoculation | i.v. or i.p. *C. parvum* |
| | Day 0 | s.c. irradiated tumour cells |
| | Day +3 | i.t. *C. parvum* |
| | | i.t. *C. parvum* mixed with irradiated tumour cells |
| | | s.c. *C. parvum* mixed with irradiated tumour cells |
| | | i.t. *C. parvum* mixed with irradiated tumour cells + local irradiation |
| Highly effective; *i.e.* tumour failed to grow in all or nearly all of the animals | Before tumour inoculation | s.c. irradiated tumour cells |
| | | s.c. *C. parvum* mixed with irradiated tumour cells |
| | Day 0 | *C. parvum* mixed with tumour inoculum |
| | | s.c. *C. parvum* mixed with irradiated tumour cells |
| | Day +3 | i.t. irradiated tumour cells |
| | | i.t. *C. parvum* mixed with viable tumour cells |

[a] Reckoned as Day 0.
[b] Abbreviations (s.c., i.v., i.t., i.p.) as in Table I.
[c] *C. parvum* refers to strain CN6134.

it is just conceivable that this is true also of the response to local injection of *C. parvum* after tumour inoculation, despite the fact that in the case of s.c. transplants their response has been found to be highly T-cell-dependent (Scott, 1974; Woodruff and Dunbar, 1975; Woodruff and Warner, 1977). The dramatic response of intracerebral transplants to s.c. injection of *C. parvum* mixed with irradiated tumour cells would seem to imply, however, that such transplants are subject to inhibition as the result of a specific immunological reaction. This is scarcely surprising, for it has long been known (Woodruff, 1960) that the status of the brain as an immunologically privileged site is by no means absolute, and is lost in the case of transplants which grow and become vascularized quickly.

The results raise the question whether it would be justifiable to set up a pilot study of local and systemic administration of *C. parvum*, as an addition to surgical resection, in patients with cerebral malignant gliomas. The extent to which the clinical therapeutic response would reflect that seen in the animal model is unpredictable. The main risk, especially with local administration of *C. parvum*, would probably be cerebral oedema. Provided the dose of *C. parvum* is kept low, however, control by standard procedures should not prove unduly difficult in patients in whom a bone flap is raised to provide surgical access.

We are indebted to Dr Keith James, Acting Head of the Department of Surgery, for making laboratory accommodation available; to the Wellcome Foundation and Dr W. McBride, respectively, for providing the *C. parvum* CN6134 and *P. freudenreichii*; to Dr N. Anderson and Mr T. Wilson for designing and casting the lead container shown in Fig. 1; and to the Wilkie, Woodruff and Hitchcock Funds of the University of Edinburgh, contributions from which made it possible to undertake this work without outside grant support.

### REFERENCES

LIKHITE, V. V. & HALPERN, B. N. (1974) Lasting Rejection of Mammary Adenocarcinoma Cell

Tumours in DBA/2 Mice with Intratumour Injection of Killed *Corynebacterium parvum*. *Cancer Res.*, **34**, 341.

McBride, W. H., Dawes, J. Dunbar, N., Ghaffar, A. & Woodruff, M. F. A. (1975) A Comparative Study of Anaerobic Coryneforms. Attempts to Correlate their Anti-tumour Activity with their Serological Properties and Ability to Stimulate the Lymphoreticular System. *Immunology*, **28**, 49.

Moore, M., Lawrence, N. & Nisbet, N. W. (1976) Inhibition of Transplanted Carcomas Mediated by BCG in Rats with a Defined Immunological Deficit. *Biomedicine* **24**, 26.

Pimm, M. V. & Baldwin, R. W. (1976) Influence of Whole Body Irradiation on BCG Contact Suppression of a Rat Sarcoma and Tumour-specific Immunity. *Br. J. Cancer*, **34**, 199.

Scott, M. T. (1974) *Corynebacterium parvum* as a Therapeutic Antitumor Agent in Mice. II. Local injection of *C. parvum*. *J. natn. Cancer Inst.*, **53**, 861.

Woodruff, M. F. A. (1960) *The Transplantation of Tissue and Organs*. Springfield: Thomas.

Woodruff, M. F. A. & Boak, J. (1966) Inhibitory Effect of Injection of *C. parvum* on the Growth of Tumour Transplants in Isogeneic Hosts. *Br. J. Cancer*, **20**, 345.

Woodruff, M. F. A. & Dunbar, N. (1973) The Effect of *Corynebacterium parvum* and Other Reticuloendothelial Stimulants on Transplanted Tumours in Mice. In *Ciba Foundation Symposium, New Series* 18, *Immunopotentiation*. Eds. G. E. W. Wostenholme & J. Knight. Amsterdam: Associated Scientific Publishers. p. 287.

Woodruff, M. F. A. & Dunbar, N. (1975) Effect of Local Injection of *Corynebacterium parvum* on the Growth of a Murine Fibrosarcoma. *Br. J. Cancer*, **32**, 34.

Woodruff, M. F. A. & Warner, N. L. (1977) The Effect of *Corynebacterium parvum* on Tumor Growth in Normal and Athymic (Nude) Mice. *J. natn. Cancer Inst.*, **58**, 111.